# ATM inhibition attenuates ferroptosis-associated injury and reduces infarct volume in experimental stroke

**DOI:** 10.70401/fos.2026.0046

**Published:** 2026-09-22

**Authors:** Han Kyu Lee, Chao-Chieh Lin, Denise E. Dunn, Yubin Chen, Ssu-Yu Chen, Douglas A. Marchuk, Scott R. Floyd, Jen-Tsan Chi

**Affiliations:** 1 Department of Molecular Genetics and Microbiology, Duke University School of Medicine, Durham, NC 27705, USA.; 2 Duke Center for Genomic and Computational Biology, Duke University, Durham, NC 27705, USA.; 3 Department of Radiation Oncology, Duke University School of Medicine, Durham, NC 27705, USA.

**Keywords:** Ferroptosis, ataxia-telangiectasia mutated, AZD1390, ischemic stroke

## Abstract

**Aims:**

Ferroptosis, an iron-dependent form of regulated cell death, contributes to neuronal injury after ischemic stroke. Because our previous cancer-cell screens identified ataxia-telangiectasia mutated (ATM) kinase, as a ferroptosis regulator, we investigated whether ATM inhibition protects neural tissue after ischemic stroke and examined determinants of therapeutic response.

**Methods:**

ATM inhibitors, including the brain-penetrant clinical compound AZD1390, were evaluated in cultured cancer and neuronal cells, oxygen-glucose-deprived rat and mouse brain slices, and permanent middle cerebral artery occlusion models using multiple inbred and Rabep2-genotype mouse strains. Cell viability, cytotoxicity, lipid peroxidation, ferroptosis-associated markers, infarct volume, and collateral vessel density were assessed. Topical AZD1390 was also tested in mice with impaired collateral networks.

**Results:**

ATM inhibition reduced ferroptosis-associated lipid oxidative injury and protected neurons in multiple preclinical stroke models. AZD1390 decreased lipid peroxidation and neuronal death, and reduced infarct volume in B6/J and B6/NJ mice, but not in BALB mice. Treatment efficacy varied among strains and was correlated with collateral vascular density. Systemic AZD1390 was likewise ineffective in Rabep2-knockout mice with deficient collateral networks. Early topical administration also reduced infarct volume in mice with limited collateral networks; however, differences in treatment timing and route preclude direct attribution to improved local drug delivery.

**Conclusion:**

ATM inhibition may attenuate ferroptosis-associated neuronal injury after ischemic stroke, while collateral vascular anatomy may influence therapeutic response. These findings support further mechanistic and translational evaluation of ferroptosis-targeting strategies in stroke.

## 1. Introduction

Ferroptosis, a regulated form of cell death driven by iron-dependent lipid peroxidation, has emerged as a critical mechanism implicated in various diseases^[1,2]^. Initially identified as a mode of cell death in RAS-mutated cancer cells, ferroptosis is characterized by iron dependency, lipid peroxidation, and mitochondrial shrinkage^[1]^. Its regulation involves key players such as glutathione peroxidase 4 (GPX4) and ferroptosis suppressor protein 1 (FSP1), which mitigate lipid peroxidation and protect against cell death^[3,4]^. Dysregulated ferroptosis has been linked to diverse pathological conditions, including tumors^[5]^, acute kidney injury^[2]^, liver disease^[6]^, CoA-related neurodegenerative disorders^[7]^, and ischemia/reperfusion injury^[2,8]^. Importantly, recent studies have identified ferroptosis as a pivotal neuronal cell death pathway triggered by ischemic injury, highlighting its relevance in ischemic stroke^[8,9]^. Ischemic stroke, caused by the disruption of blood flow to the brain, results in oxidative stress, iron overload, and the accumulation of reactive oxygen species (ROS), all of which synergistically induce ferroptotic cell death^[8,9]^. Recent work has further situated this lipid peroxidation cascade within a broader program of post-ischemic metabolic reprogramming, in which subacute lipid peroxidation emerges as a principal driver of neuronal ferroptosis^[10]^. This process exacerbates neuronal damage and worsens stroke outcomes, highlighting ferroptosis as a potential therapeutic target. Despite its significance, the underlying regulatory mechanisms of ferroptosis in stroke remain obscure.

Although inhibiting ferroptosis presents a promising therapeutic approach for ischemic stroke, currently established ferroptosis inhibitors are not suitable for clinical application. Ferroptosis has been most extensively characterized in cancer, where unbiased screening approaches are most tractable and where the core regulatory machinery was originally defined^[1,5]^. To uncover key regulators of ferroptosis, we previously conducted forward kinome screens for modifiers of ferroptotic responses in breast cancer cell lines, highlighting ataxia telangiectasia mutated (ATM) kinase as a top candidate^[11]^. Our findings demonstrate that ATM inhibition reduces labile iron levels by inducing the expression of iron-sequestering proteins such as ferritin and ferroportin, mediated through metal regulatory transcription factor 1 (MTF1)^[11]^. Another independent study has further validated this result^[12]^, and a separate review^[13]^ provides justification for combining ionizing radiation with ferroptosis-based therapeutics.

However, the relevance of ATM in ferroptosis-associated disease processes beyond cancer remains unknown. Given the availability of numerous ATM inhibitors developed for preclinical and clinical studies, we propose that ATM inhibition represents a promising translational approach to mitigating ferroptosis in ischemic stroke and other ferroptosis-associated diseases. With the challenge of reaching ischemic brain tissues through the blood-brain barrier, we noted that AZD1390 is an orally bioavailable and brain-penetrant ATM inhibitor that shows efficacy against intracranial glioma models *in vivo*^[14]^ and crosses the blood-brain barrier in humans^[15]^. It is currently being evaluated in clinical trials (NCT03423628). Thus, the successful application of AZD1390 may have significant translation potential. Notably, AZD1390 has recently been reported to reduce infarct volume and improve neurological deficits in experimental stroke^[16]^, an effect attributed to attenuated microglia-mediated neuroinflammation. Whether ATM inhibition also engages the ferroptotic cascade in ischemic neurons, and what determines whether a systemically administered ATM inhibitor reaches the ischemic territory, have not been addressed.

To bridge the gap between the previous cancer cell line study and future translation, we investigated the therapeutic potential of ATM inhibition by AZD1390 using various preclinical *ex vivo* and *in vivo* models of ischemic stroke. Here, we show that ATM inhibition using AZD1390 not only limits ferroptosis *in vitro* but also significantly reduces neuronal cell death in both *ex vivo* organotypic brain slice models and *in vivo* mouse models of ischemic stroke. AZD1390 treatment reduced infarct volume, and this protection was accompanied by reduced ferroptosis-associated lipid peroxidation. Additionally, we reveal that there is significant variation in AZD1390 efficacy among different inbred mouse strains, and this variation is associated with the extent of the collateral vascular network, which critically affects drug delivery to the ischemic territory. These results highlight not only the molecular and translational potential of AZD1390, but also the determinants of therapeutic response, facilitating individualized stroke treatments. These results underscore the therapeutic potential of targeting ferroptosis pathways as a physiologically relevant and promising translation pathway for ischemic stroke in humans.

## 2. Materials and Methods

### Sex as a biological variable.

Sex as a biological variable was considered in the design of this study. Both male and female animals were included for all experimental groups to ensure that findings are not sex-biased. In the larger cohorts used for the oral AZD1390 infarct volume studies in the inbred mouse strains and in *Rabep2* mice, group sizes were determined by litter availability during breeding, and sex ratios therefore deviated modestly from 1:1; vehicle-treated *Rabep2* Het mice comprised 4 males and 7 females, vehicle-treated *Rabep2* KO mice comprised 5 males and 7 females, and B6/J vehicle mice comprised 7 males and 5 females. The groups contributing to the topical AZD1390 study each comprised three males and three females.

### Animals.

All inbred mouse strains were obtained from the Jackson Laboratory (Bar Harbor, ME), and then bred locally to generate mice used in all experiments. Sprague-Dawley (SD) rats for *ex vivo* brain slice culture experiments were obtained from Charles River (Wilmington, MA). 12 ± 1-week-old mice (both male and female animals) were used for permanent middle cerebral artery occlusion (pMCAO) and postnatal day 7 mice and rats were used for *ex vivo* brain slice culture.

### Cell culture.

MDA-MB-231 cells were obtained from the Cell Culture Facility at Duke University. HT-22 mouse hippocampal neuronal cells were a gift from Dr. Jiahua Xie (North Carolina Central University, Durham, NC, USA). Before freezing, the cell lines were authenticated via STR DNA profiling and confirmed to be free of mycoplasma contamination. The cells were maintained for less than six months and cultured in DMEM medium (GIBCO-11995) supplemented with 10% heat-inactivated fetal bovine serum (ThermoFisher #10082147) and antibiotics (streptomycin (10,000 U/mL) and penicillin (10,000 U/mL); ThermoFisher #15140122). Cultures were incubated at 37 °C in a humidified atmosphere with 5% CO_2_.

### AZD1390 preparation.

Pharmaceutical-grade AZD1390 for *in vivo* studies was obtained directly from AstraZeneca and was prepared as recommended by the manufacturer’s formulation and doses for *in vivo* mouse studies. Briefly, a dose of 20 mg/kg was suspended in a vehicle consisting of 0.5% hydroxypropyl methylcellulose (HPMC) and 0.1% Tween 80 in water. This suspension was either stirred or rotated overnight at room temperature in the absence of light. Freshly prepared drug formulations were used within 2 days for *in vivo* experiments.

### Chemicals.

All chemicals, including drugs and inhibitors, were used as previously described. Briefly, erastin (Bio-Techne #5499), ferrostatin-1 (Cayman #17729), deferoxamine (Sigma D9533), liproxstatin-1 (Cayman #17730), KU55933 (Selleckchem #S1092), and KU60019 (Selleckchem #S1570) were prepared as stock solutions in DMSO. For experiments, these stock solutions were diluted 1:1,000 in culture media to prepare working solutions.

### Cell viability and cytotoxicity assays.

Cell viability was assessed using the CellTiter-Glo luminescent viability assay (Promega) as recommended by the manufacturer’s instructions. In brief, 15 μL of the CellTiter-Glo substrate was added to wells containing cells in 100 μL of culture medium in a 96-well plate. The plate was shaken for 10 minutes, and the luminescent signal was quantified using a chemiluminescence plate reader. Cytotoxicity was evaluated by the CellTox Green assay (Promega), which was diluted 1:1,000 in culture medium, and fluorescence was measured.

### Quantitative PCR.

mRNA levels were measured by quantitative PCR (qPCR) as previously described. To quantify mRNA levels of *xCT* and *PTGS2*, rat brain slices were treated with or without OGD, followed by either Ferrostatin-1 (Fer-1) or AZD1390. All samples were analyzed in triplicate, and an additional assay targeting endogenous *Gapdh* was included to normalize for input cDNA template quantity. Primer sequences for rat included:

*xCT*: Forward 5’- TAC CTG CAG GGC AAT GTG AG-3’, Reverse 5’- AGT ATG CCC TTG GGG GAG AT-3’; *PTGS2*: Forward 5’-TGA CTG TAC CCG GAC TGG AT-3’, Reverse 5’- CAT GGG AGT TGG GCA GTC AT -3’; *Gapdh*: Forward 5’-GCG AGA TCC CGC TAA CAT CA-3’, Reverse 5’-CTT GCG GTC CCC ACA TAC C-3’

### Lipid peroxidation assay.

Lipid peroxidation was evaluated using the C11-BODIPY dye (ThermoFisher, D3861). Cells were treated with either vehicle or erastin with/without various doses of AZD1390 for 16 hours. Subsequently, a 10 μM solution of C11-BODIPY was added to the culture medium for 1 hour at 37 °C. The cells were harvested, washed, and resuspended in PBS with 1% BSA. C11-BODIPY oxidation was quantified using the mean fluorescence intensity of the oxidized probe in the FITC/Alexa Fluor 488 channel using flow cytometry (FACSCanto II, BD Biosciences).

### Western blot analysis.

Protein samples were collected from rat brain slices (Figure S2) or from mouse brain tissues following ischemic stroke induction and treatment with either vehicle or AZD1390. Briefly, the protein concentrations were determined via the BCA assay (ThermoFisher, #23227) after lysing pulverized mouse brain samples in RIPA buffer. Approximately 20 μg of protein per sample was resolved on 8% SDS-PAGE gels, transferred to PVDF membranes, blocked with 5% non-fat milk in 1 × TBST, and incubated overnight at 4 °C with primary antibodies. Antibodies used included MDA (1:1,000, ab27642, Abcam); GPX4 (1:1,000, ab125066, Abcam); ACSL4 (1:1,000, sc-365230, Santa Cruz); GAPDH (1:2,000, sc-25778, Santa Cruz). All bands of MDA-modified proteins in each lane were included for quantification by ImageJ.

### Preparation of brain slices and oxygen glucose deprivation (OGD).

Preparation of cortical brain slice explants was performed as previously described^[7,17]^. Coronal brain slices were prepared from P7 rats and mice. Under sterile conditions, brains were dissected and cut into 250-μm-thick coronal slices on a vibratome in chilled culture medium containing 15% heat-inactivated horse serum, 10 mM KCL, 10 mM HEPES, 100U/mL penicillin/streptomycin, 1 mM MEM sodium pyruvate, and 1 mM L-glutamine in Neurobasal A supplemented with 1 μM MK-801.

Brains were divided into hemi-coronal slices. For OGD, slices were suspended at 34 °C for 4.5 minutes in glucose-free N_2_-bubbled artificial CSF containing 124 mM NaCl, 25.7 mM NaCO_3_, 4.9 mM KCL, 1.2 mM KPO_4_, 3.1 mM CaCl_2_, 1.3 mM MgSO_4_ (pH 7.0). Brain slices were plated into 12-well plates in an interface configuration on top of solid culture medium prepared by the addition of 0.5% agarose. After explanting the brain slices, plates were placed for recovery at 30 °C for 30 minutes in a humidified incubator under 5% CO_2_. Gold particles with a diameter of 1.6 μm coated with plasmids expressing YFP in G-Wiz vector were introduced into the brain slices by biolistic transfection using a Helios Gene Gun (Bio-Rad). Slice cultures were maintained at 37 °C for 24 hours in a humidified incubator under 5% CO_2_. Viable neurons were scored by YFP morphology under fluorescent stereoscope visualization.

For each independent experiment, hemi-coronal brain slices obtained from 12 animals were mixed and randomly distributed across the treatment conditions. Twelve individual slices were analyzed per condition in each experiment. Three independent experiments were performed, resulting in 36 slice-level observations per condition. During routine processing, some slices did not yield analyzable measurements because of physical damage during preparation or culture or insufficient YFP labeling to permit reliable neuronal scoring. Consequently, the final number of evaluable slices varied among conditions. Neuronal viability was measured separately in each slice. Because the slices were randomized during allocation, the source animal associated with each individual slice was not retained, preventing retrospective modeling of within-animal clustering.

### Permanent middle cerebral artery occlusion and AZD1390 treatment.

Focal cerebral ischemia was induced by direct permanent occlusion of the distal MCA as previously described^[18–20]^. Briefly, adult mice were anesthetized with ketamine (100 mg/kg) and xylazine (5 mg/kg), and then 0.5% bupivacaine (5 mg/mL) was also administered by injection at the incision site. The right MCA was exposed by a 0.5 cm vertical skin incision midway between the right eye and ear under a dissecting microscope. After the temporalis muscle was split, a 2 mm burr hole was made with a high-speed micro drill at the junction of the zygomatic arch and the squamous bone through the outer surface of the semi-translucent skull. The MCA was clearly visible at the level of the inferior cerebral vein. The inner layer of the skull was removed with fine forceps, and the dura was opened with a 32-gauge needle. While visualizing under an operating microscope, the right MCA was electrocauterized. The cauterized MCA segment was then transected with microscissors to verify permanent occlusion. The surgical site was closed with 6–0 sterile nylon sutures, and 0.5% bupivacaine was applied. The temperature of each mouse was maintained at 37 °C with a heating pad during the surgery, and then the mouse was placed in a recovery chamber (set temperature 37 °C) until the animal was fully recovered from the anesthetic. Two hours after pMCAO, animals received a 20 mg/kg dose of AZD1390 via oral gavage using a flexible feeding tube (Instech, Plymouth Meeting, PA). For topical application, 40 μL of a 26.17 mM (12.5 mg/mL) AZD1390 solution was applied to the exposed cortical surface 15 minutes after pMCAO. The total volume was delivered sequentially in four separate 10 μL drops, ensuring each drop was completely absorbed into the infarct area before the next application. Mice were then returned to their cages and allowed free access to food and water in an air-ventilated room maintained at an ambient temperature of 25 °C.

### Infarct volume measurement.

Cerebral infarct volumes were measured 72 hours after distal permanent MCA occlusion as previously described^[18–20]^. Seventy-two hours after pMCAO surgery, the animals were euthanized, and the brains were carefully removed. The brains were placed in a brain matrix, chilled at −80 °C for 4 min to slightly harden the tissue, and then sliced into 1 mm coronal sections. Each brain slice was placed in 1 well of a 24-well plate and incubated for 20 min in a solution of 2% 2,3,5-triphenyltetrazolium chloride (TTC) in PBS at 37 °C in the dark. The sections were then washed once with PBS and fixed with 10% PBS-buffered formalin at 4 °C. Then 24 hours after fixation, the caudal face of each section was scanned using a flatbed color scanner. The scanned images were used to determine infarct volume. Image-Pro software (Media Cybernetics, Inc., MD) was used to calculate the infarcted area of the hemisphere to minimize error introduced by edema. The total infarct volume was calculated by summing the individual slices from each animal.

### Collateral vessel density measurement.

The collateral vessel phenotype was measured at P21 as previously described^[18–20]^. Mice were anesthetized with ketamine (100 mg/kg) and xylazine (5 mg/kg), and the ascending thoracic aorta was cannulated. The animals were perfused with freshly made buffer (1 mg/mL adenosine, 40 μg/mL papaverine, and 25 mg/mL heparin in PBS) to remove the blood. The pial circulation was then exposed after removal of the dorsal calvarium and adherent dura mater. The cardiac left ventricle was cannulated and a polyurethane solution with a viscosity sufficient to minimize capillary transit (1:1 resin to 2-butanone, PU4ii, VasQtec) was slowly infused; cerebral circulation was visualized under a stereomicroscope during infusion. The brain surface was topically rinsed with 10% PBS-buffered formalin and the dye solidified within 20 min. After post-fixation with 10% PBS-buffered formalin, pial circulation was imaged. All collaterals interconnecting the anterior- and middle cerebral artery trees of both hemispheres were counted.

### Study design and methodological rigor.

All experimental group sizes were established based on prior experience and statistical power consideration. Both male and female animals were included across the *in vivo* studies to account for sex as a biological variable. Group allocations included either equal representation (e.g., *n* = 6 total per group, consisting of 3 males and 3 females) or approximately equal, balanced distributions across sexes in larger groups (e.g., up to *n* = 17 per group); exact sex distributions for each group are detailed in the Supplemental Table. For *in vitro* and *ex vivo* assays, experiments were performed with a minimum of *n* = 3 independent biological replicates per group. Animals were randomly assigned to the treatment or control group prior to procedures. Investigators were blinded to experimental conditions, treatment arms, and surgical groups during data collection and analysis. Pre-established exclusion criteria were defined for technical failures or non-completion of experimental protocol. However, no animals were excluded from the final analysis, and all raw data were fully reported. All units and chemical concentrations throughout the manuscript were thoroughly corrected for accuracy.

### Statistical analyses.

All statistical analyses were conducted using GraphPad Prism (GraphPad Software, La Jolla, CA). The distribution of each dataset was evaluated using the Shapiro-Wilk test to assess normality. Selection of statistical tests was guided by these distributional assessments. Datasets in which all comparison groups satisfied normality assumptions were analyzed using parametric methods, whereas datasets in which one or more groups deviated from normality were analyzed using non-parametric approaches. For pairwise comparisons between two independent groups with normally distributed data and comparable variances, two-tailed Student’s t-tests were applied. When more than two groups were compared under conditions of normality, one-way analysis of variance (ANOVA) was used, followed by Dunnett’s post hoc test to evaluate differences relative to the control group. For datasets that did not meet normality assumptions, group differences were assessed using the Kruskal-Wallis test, with Dunn’s multiple comparisons applied for post hoc analyses. For experiments involving two independent factors, two-way ANOVA was performed only when normality criteria were met. When these criteria were not satisfied, factorial ANOVA was not used; instead, simple-effects analyses were conducted by comparing treatment conditions within each level of the second factor using Mann-Whitney U tests, with appropriate adjustments for multiple comparisons. Data from parametric analyses are presented as mean ± standard error of the mean (SEM). For non-parametric analyses, results are displayed as individual data points with the median and interquartile range (IQR). Data visualization was selected to align with the statistical methods employed.

## 3. Results

### 3.1 ATM inhibitors protect against erastin-induced ferroptosis *in vitro*

To validate our previous finding that inhibiting ATM protects against cell death induced by erastin, a canonical ferroptosis inducer^[11]^, we tested the established ferroptosis inhibitor, liproxstatin-1 (LPT1), as well as ATM inhibitors, KU55933 and KU60019. As expected, both LPT1 and ATM inhibitors significantly increased cell viability following erastin-induced ferroptosis (Figure 1a). These results confirm our previous observation that ATM plays a critical role in the ferroptosis pathway^[11]^. AZD1390 is an orally bioavailable and brain-penetrant ATM inhibitor being clinically evaluated^[14,15]^ (NCT03423628). Given the translation potential of AZD1390, a brain-penetrating ATM inhibitor, we further investigated its potential in protecting against ferroptosis. We found that AZD1390 dose-dependently reduced erastin-induced cell death (Figure 1b). We also measured membrane rupture and cytotoxicity to assess its effects on membrane integrity, observing a dose-dependent decrease in cell membrane rupture with AZD1390 treatment (Figure 1c). Additionally, erastin-induced lipid peroxidation, a hallmark of ferroptosis, was significantly reduced by AZD1390 treatment as measured by C11-BODIPY and flow cytometry (Figure 1d,e). Similarly, in HT-22, a hippocampal neuronal cell line, we also found that AZD1390 mitigated erastin-induced ferroptosis by lipid peroxidation (Figure S1) and cell viability (Figure S1). These findings strongly support our hypothesis that inhibiting ATM prevents cell death by inhibiting ferroptosis.

### 3.2 AZD1390 protects against neuronal cell death in *ex vivo* brain slice models of stroke

We initially used MDA-MB-231 cells to reproduce and validate the ferroptosis-protective role of ATM inhibition established in prior studies. This allowed direct benchmarking against well-characterized systems. After demonstrating the ability of ATM inhibitors to protect against ferroptosis in cancer cell lines, we subsequently wished to extend this finding to non-cancer settings using various physiologically relevant models, such as *ex vivo* cortical neuronal cell death induced by ischemic stroke (Figure 2), thereby extending findings to central nervous system contexts. To assess the pharmacological effectiveness of ATM inhibitors, we employed organotypic brain slice culture models of stroke by exposing them to OGD that mimics oxygen and glucose deprivation during ischemic stroke. Treatment of brain slices from SD rats following OGD with three different ATM inhibitors consistently protected against neuronal cell death (Figure 2a,b,c,d). AZD1390 rescued neuronal cell viability in this assay, although its effectiveness was somewhat lower compared to other ATM inhibitors. However, given that AZD1390 is a clinical compound demonstrating benefits in promoting axon regeneration and functional recovery following spinal cord injury^[21]^, we focused subsequent studies on this compound. To verify whether AZD1390 rescues neuronal cell viability via ferroptosis, we determined transcription levels of the following ferroptosis markers: Solute Carrier Family 7 Member 11 (*SLC7A11*) (Figure 2e) and prostaglandin-endoperoxide synthase 2 (*PTGS2*) (Figure 2f) from rat brain slices after transient OGD treatment. Both *xCT* and *PTGS2* transcript levels were elevated in brain slices following OGD treatment. Importantly, AZD1390 reduced their induction under OGD in a dose-dependent manner, mirroring the effects of the ferroptosis inhibitor, ferrostatin-1 (Fer-1) (Figure 2e,f). However, OGD treatment did not alter the protein levels of two canonical ferroptosis markers, GPX4 and ACSL4 (Figure S2). The absence of a change in total GPX4 or ACSL4 protein does not exclude ferroptosis, as ferroptosis is governed primarily by GPX4 enzymatic activity and ACSL4-dependent phospholipid remodeling rather than by changes in the total abundance of these proteins^[3,22]^; together with the reduced lipid peroxidation and the protection afforded by the mechanistically distinct ferroptosis inhibitors ferrostatin-1, liproxstatin-1, and deferoxamine, these findings indicate that AZD1390 limits ferroptotic injury at the level of lipid peroxidation. We also evaluated the effectiveness of AZD1390 in reducing neuronal death in cortical brain slices from C57BL/6J (B6/J) mice and observed that, as with the rat brain slices, AZD1390 treatment led to increased cell viability (Figure 2g). Together, the protection produced by ferrostatin-1 and multiple ATM inhibitors, accompanied by reduced lipid peroxidation and ferroptosis-associated measurements, is consistent with attenuation of ferroptosis-associated neuronal injury. However, these pharmacological and correlative measurements do not establish direct ATM target engagement or exclude contributions from other forms of ischemic cell injury.

### 3.3 Brain-penetrant AZD1390 reduces infarct volume after pMCAO in an *in vivo* stroke model

To further extend these findings *in vivo*, we induced ischemic stroke by surgical occlusion of the middle cerebral artery in commonly used inbred mouse strains, B6/J and BALB/cJ (BALB), including a B6/J substrain, C57BL/6NJ (B6/NJ), and determined the effects of administered AZD1390. Mice received 20 mg/kg of AZD1390 via oral gavage two hours after permanent MCAO, and infarct volume was measured 72 hours post-pMCAO (Figure 3a). This dosing strategy follows a prior study demonstrating AZD1390’s brain penetration in mouse brains^[14]^, and delivery at two hours after surgical occlusion is consistent with a translationally feasible therapeutic approach that better reflects clinical context but presents a more rigorous test. AZD1390, when compared with vehicle control, was found to significantly reduce infarct volume in both mouse strains, B6/J and B6/NJ, with decreases of 55.6% in B6/J and 37.1% in B6/NJ (Figure 3b,c). However, no significant reduction in infarct volume was observed in BALB mice (Figure 3c), suggesting a strain-dependent response to AZD1390. To explore the underlying mechanism of protection in drug-responsive strains, we investigated malondialdehyde (MDA) levels, a byproduct of lipid peroxidation during ferroptosis. Ischemic mice showed dramatically reduced levels of MDA after AZD1390 treatment compared to vehicle-treated mice (Figure 3d,e). Collectively, AZD1390 treatment was associated with reduced infarct volume and lower MDA-modified protein abundance. Because MDA is a general indicator of lipid oxidative damage, these results are consistent with reduced lipid-peroxidation-associated injury but do not directly establish ferroptosis as the mechanism of protection.

### 3.4 Brain collateral vascular anatomy significantly impacts the therapeutic effect of AZD1390

As shown in Figure 3c, while AZD1390 provided a protective effect in inbred mouse strains B6/J and B6/NJ, there was no effect in the BALB strain. Among the many potential phenotypic differences that might explain their differences in drug efficacy, these strains exhibit a well-described and genetically determined difference in brain collateral vessel anatomy^[18–20]^. We repeated these anatomic measurements for the mice used in this study by analyzing the number of collateral vessel connections between the anterior cerebral artery (ACA) and the middle cerebral artery (MCA). As previously shown, while B6/J and B6/NJ mice exhibited a high and similar number of collateral connections, BALB mice showed a dramatic reduction (Figure 4a,b,c). This suggests that the cerebral collateral vascular network may be critical for delivering AZD1390 to ischemic regions of the brain. To investigate whether an extensive collateral vessel network is essential for the therapeutic efficacy of AZD1390, we employed *Rabep2* (RAB GTPase-binding effector protein) knockout (KO) mice held on a B6/J background^[23]^. *Rabep2* KO mice were used to genetically model reduced collateral vessel density on a uniform B6/J background, allowing for a direct assessment of the relationship between collateral anatomy and drug efficacy. Our use of *Rabep2* KO here is specifically to dissect the role of vascular access in AZD1390 efficacy, not to model its therapeutic effect. As previously described^[23,24]^, we confirmed that *Rabep2* KO mice exhibit a significantly reduced extent of collateral vessel connections, similar to that of the inbred strain BALB. In contrast, *Rabep2* wild-type (WT) and heterozygous (Het) mice display extensive collateral vessel networks, similar to those of strains B6/J and B6/NJ. We next evaluated the efficacy of AZD1390 in *Rabep2* KO mice. As observed in B6/J and B6/NJ, AZD1390 significantly reduced infarct volume in *Rabep2* WT (35.9% reduction) and Het (45.0% reduction) mice compared to their respective vehicle-treated controls (Figure 4d). By contrast, AZD1390 had no effect on infarct volume in *Rabep2* KO mice (Figure 4d). These results support our hypothesis that a sufficiently dense collateral vessel network is essential for effective delivery of AZD1390 to the ischemic brain territory, and, consequently, for its protective effect.

### 3.5 Topical application of AZD1390 reduces infarct volume in mice with impaired collateral vascular networks

The lack of AZD1390 efficacy in *Rabep2* KO and BALB mice, both of which exhibit significantly diminished collateral vessel networks, suggests that poor drug delivery to the ischemic territory may underlie the observed resistance to treatment. However, these findings raise the possibility that strain-specific differences in pharmacodynamics or intrinsic resistance mechanisms could also contribute to the difference in efficacy. To distinguish between these possibilities, we sought to bypass the vascular delivery route altogether by applying AZD1390 directly to the infarcted brain region. This approach allows us to isolate the contribution of vascular delivery from other potential mechanisms of resistance and directly test whether localized drug availability is sufficient to restore therapeutic benefit in mice with impaired collateral circulation. We applied 40 μL of AZD1390 (12.5 mg/mL, 26.17 mM; 0.5 mg total) topically to the exposed cortical surface 15 minutes after pMCAO, before closing the surgical area, and measured infarct volume 72 hours post-surgery (Figure 5a). Under this experimental model, we assessed the effect of AZD1390 in both BALB and *Rabep2* KO mice. Topical AZD1390 application significantly reduced infarct volume in both BALB (28.8% reduction) and *Rabep2* KO (35.2% reduction) compared to their respective vehicle-treated controls (Figure 5b,c). These findings indicate that the lack of therapeutic effect observed in systemic administration may be attributable to impaired drug delivery to the ischemic territory rather than strain-specific intrinsic resistance.

## 4. Discussion

Our previous study identified ATM inhibition as a promising approach to suppress ferroptosis, using cancer cell lines as a model system. While that work established proof-of-concept for targeting ATM to modulate ferroptosis, its translational potential in non-cancer disease contexts had not been explored. Building upon these findings, the current study moves beyond cancer cell lines to demonstrate the efficacy of a brain-penetrant clinical ATM inhibitor, AZD1390, in reducing neuronal damage in both *ex vivo* and *in vivo* models of ischemic stroke. This progression from mechanistic insights *in vitro* to preclinical models of neurological injury highlights the translational relevance of ATM inhibition and positions AZD1390 as a compelling candidate for future clinical investigation in stroke therapy.

The cellular context differs substantially between these models. We first identified the role of ATM in regulating ferroptosis from a kinome screen in MDA-MB-231 cells^[11]^, a transformed, highly proliferative breast cancer cell line with oncogenic and metabolic adaptations that can alter iron handling, lipid metabolism, antioxidant defenses, and ATM signaling, whereas neurons are post-mitotic cells with high oxidative demand and polyunsaturated fatty acid-rich membranes and are further influenced by glial and vascular interactions in intact brain tissue^[1,2,8,9,11]^. Thus, protection observed in cancer cells cannot be assumed to translate directly to non-neoplastic neural tissue or to arise through identical downstream mechanisms. Although HT-22 is an immortalized neuronal cell line, the concordant effects of AZD1390 in HT-22 cells, organotypic rat and mouse brain slices, and mouse pMCAO models support a shared, tissue-spanning role for ATM in ferroptotic injury. Direct side-by-side mechanistic comparisons between cancer cells and normal neuronal cells will be important to define which downstream components are conserved and which are context-dependent.

Previous studies have reported that ATM inhibition protects against ischemic brain injury in the transient middle cerebral artery occlusion (tMCAO) model^[16,25]^. These studies also reported improved neurological outcomes following ATM inhibitor treatment compared to vehicle controls^[16,25]^. While tMCAO models more closely mimic clinical stroke involving reperfusion, we selected the permanent MCAO (pMCAO) model in this study due to its superior reproducibility and suitability for genetic comparisons across inbred and mutant mouse strains. As our primary objective was to elucidate ferroptosis as a key mechanism of neuronal cell death in ischemic stroke and to evaluate ATM inhibitors as a potential therapeutic strategy, our study does not repeat tMCAO or behavioral assessment. However, future studies using tMCAO and incorporating long-term neurological outcome measures will be essential to validate the translational efficacy of AZD1390 in a broader clinical context, ideally alongside the neuromodulatory and rehabilitative strategies now being developed to support post-stroke functional recovery^[26]^. While ferroptosis has been previously implicated in various pathological conditions, including cancer and acute kidney injury^[2]^, our findings are consistent with a contributing role in ischemic stroke-induced neuronal damage. By demonstrating that ATM inhibitors, particularly the brain-penetrant AZD1390, mitigate neuronal cell death and infarct volume in both *ex vivo* and *in vivo* models, we link ischemic stroke pathology to ferroptosis-associated lipid oxidative injury. The reduced induction of ferroptosis-associated transcripts, such as xCT and PTGS2, is consistent with, but does not by itself establish, a ferroptosis-dependent mechanism of protection. These results extend our understanding of the molecular events^[14,15]^, making it a strong candidate for translational application. AZD1390 is being evaluated in a clinical trial (NCT03423628). Additionally, we identify significant variation in the therapeutic efficacy of AZD1390 among different mouse strains with ischemic stroke. To minimize variability across multiple experiments with inbred strains and *Rabep2* KO mice, we employed the permanent MCAO model, which produces a more reproducible infarct volume, allowing us to more accurately evaluate the therapeutic impact of AZD1390. Through additional analysis, we found that this variation primarily results from differences in collateral vascular density. In mouse strains with limited collateral vessel connections (inbred strain BALB and *Rabep2* KO mice), the protective effect of AZD1390 is diminished, likely due to impaired drug delivery to the ischemic territory. Notably, the direct topical application of AZD1390 to the cortical surface successfully restored its protective effect, indicating that the differences in efficacy are unlikely to result from intrinsic resistance to AZD1390. These findings highlight both the importance of vascular access for systemically delivered therapies and the potential for alternative delivery strategies. Nevertheless, several limitations should be acknowledged. While our preclinical models provide robust proof of concept, stroke is a heterogeneous and multifactorial condition, and further studies are needed to assess the efficacy of ATM inhibition across diverse stroke subtypes and in larger animal models. While our current studies highlight the importance of collateral vascular networks in therapeutic efficacy, there may be additional determinants that need to be further identified. The long-term safety of systemic ATM inhibition, particularly in the context of neuronal recovery and immune response, remains to be evaluated. Moreover, while our data implicate ferroptosis as a key driver of ischemic injury, additional work is required to clarify the interplay between ferroptosis and other regulated cell death pathways in stroke pathology. Lipid-peroxidation measurements also require cautious interpretation because lipid ROS can occur during other regulated cell-death processes, and C11-BODIPY oxidation or MDA abundance alone does not establish ferroptosis. More specific approaches, such as functional rescue and analysis of oxidized phospholipid species, are needed to distinguish ferroptosis-associated lipid peroxidation from general oxidative injury^[27]^. Finally, the timing of drug administration differed between our systemic and topical experiments: oral AZD1390 was given 2 hours after pMCAO (Figure 3), whereas topical AZD1390 was applied 15 minutes after pMCAO (Figure 5). Although we interpret the restored efficacy of topical delivery in BALB/c and *Rabep2* KO mice as evidence that direct vascular bypass overcomes the delivery barrier imposed by a poor collateral network, we cannot exclude the possibility that the earlier administration time of the topical route contributes to its efficacy through an expanded therapeutic window. Definitively distinguishing these possibilities would require applying the topical treatment at the same 2-hour time point used for oral gavage, and this timing difference should therefore be taken into account when interpreting the vascular-dependency hypothesis.

In summary, this study builds on foundational mechanistic work in cancer models to show that ATM inhibition reduces ischemic neuronal injury and infarct volume in preclinical stroke models. Our findings provide a strong basis for further translational investigation of ATM inhibition as a potential therapeutic strategy in ischemic stroke.

Several mechanistic limitations should be considered. Although three pharmacologically distinct ATM inhibitors protected neurons in the brain-slice model, direct ATM target engagement was not measured in neuronal or ischemic brain tissues, and neuronal ATM was not genetically manipulated. Furthermore, PTGS2 induction and MDA-modified proteins are not specific to ferroptosis, while C11-BODIPY oxidation can reflect lipid-reactive oxygen species arising in other forms of cell injury. Accordingly, these measurements should be interpreted as evidence of ferroptosis-associated lipid oxidative injury rather than definitive demonstration of ATM-dependent neuronal ferroptosis. Future studies measuring neuronal ATM signaling, incorporating genetic ATM perturbation and ferroptosis-specific rescue *in vivo*, and analyzing oxidized phospholipid species will be required to establish the proposed mechanism more directly.

The present *in vivo* studies measured infarct volume 72 hours after pMCAO but did not include behavioral, neurological, or long-term recovery assessments. Therefore, reduced infarct volume should be interpreted as attenuation of acute structural brain injury and not as evidence of functional recovery. Future studies incorporating neurological scoring, sensorimotor testing, and longer-term outcomes will be needed to determine whether the reduction in infarct volume translates into functional benefit.

## 5. Conclusion

This study implicates ferroptosis-associated lipid oxidative injury in neuronal cell death after ischemic stroke, highlighting ATM inhibition, particularly with the brain-penetrant AZD1390, as a promising therapeutic strategy. By showing that ATM inhibitors reduce neuronal cell death and infarct volume in both *ex vivo* and *in vivo* models, this research supports further investigation of ferroptosis as a therapeutic target in stroke. The efficacy of AZD1390, an orally bioavailable and brain-penetrating clinical compound, underscores its translational relevance. These findings pave the way for the development of ferroptosis-targeting therapies to mitigate neuronal injury and improve clinical outcomes for stroke patients, offering a pathophysiologically grounded approach with clinical feasibility.

## Supplementary Material

Supplementary materials

The supplementary material for this article is available at: Supplementary materials.

## Figures and Tables

**Figure 1.**
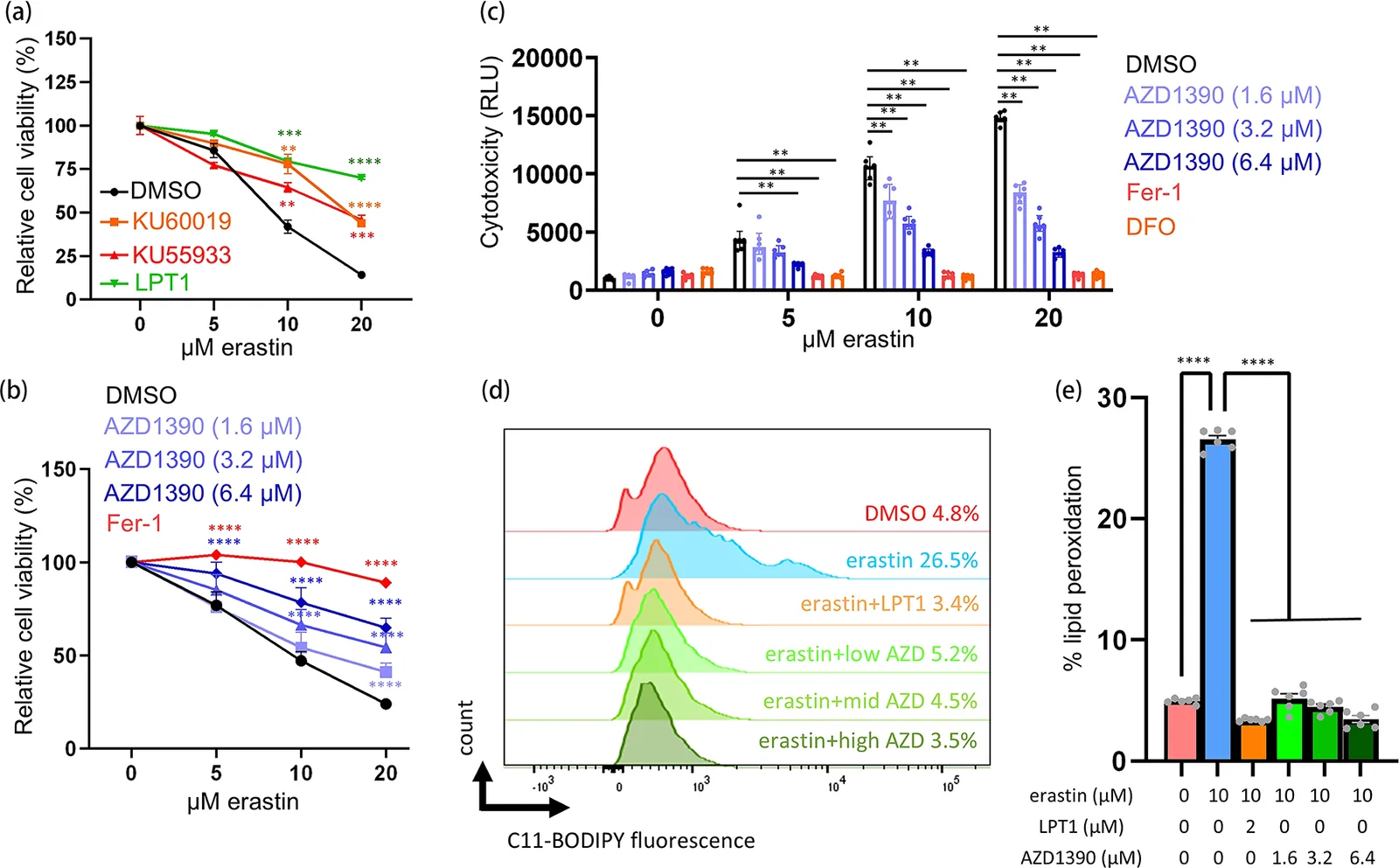
AZD1390 protects against erastin-induced ferroptosis in MDA-MB-231 cells. (a) ATM inhibitors protect MDA-MB-231 cells against ferroptosis. MDA-MB-231 cells were treated with increasing doses of erastin (24 h), either alone or in combination with liproxstatin-1 (LPT1, 2 μM), KU55933 (5 μM), and KU60019 (1 μM). Cell viability was quantified by CellTiter-Glo assay; (b, c) AZD1390 reduced erastin-induced ferroptosis in MDA-MB-231 cells in a dose-dependent manner. After 24 hours of incubation, cell viability and cytotoxicity were determined by CellTiter-Glo assay (b) and CellTox Green assay (c), respectively. Canonical ferroptosis inhibitors: Fer-1; DFO; (d, e) Erastin-induced lipid peroxidation (10 μM, 18 hours) in MDA-MB-231 cells was reduced by AZD1390; Lipid peroxidation was assessed by C11-BODIPY staining (d) and quantified as the percentage of lipid peroxidation-positive cells (e). (a, b, and e) Data represent the mean ± SEM. (a, b) 2-way ANOVA and Dunnett’s multiple comparisons. (e) One-way ANOVA followed by Dunnett’s multiple comparison test. (c) Non-parametric Mann-Whitney tests with correction for multiple comparisons. Data represents the median ± IQR. (a-e) *n* = 6 independent biological replicates. *p < 0.05; **p < 0.01; ***p < 0.001, ****p < 0.0001. MDA: malondialdehyde; ATM: ataxia-telangiectasia mutated; LPT1: liproxstatin-1; Fer-1: Ferrostatin-1; DFO: Deferoxamine; SEM: standard error of the mean; ANOVA: analysis of variance; IQR: interquartile range.

**Figure 2.**
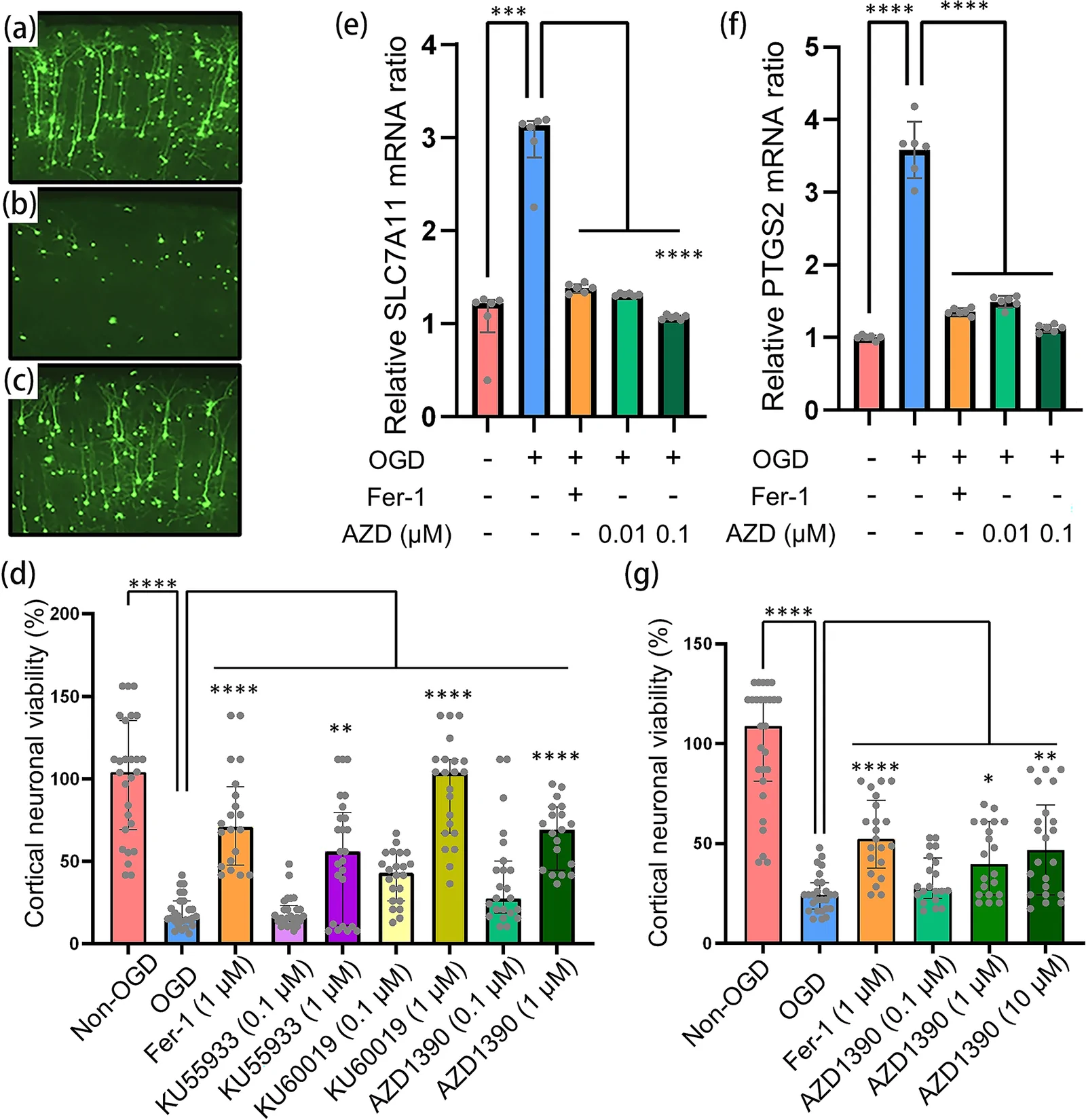
AZD1390 increases neuronal cell viability in the cortical brain slice ischemia assay. (a, b, and c) Representative images showing YFP-transfected cortical pyramidal neurons in rat brain slices under non-OGD control conditions (a), after 4.5 minutes of OGD (b), and after 4.5 minutes of OGD followed by Fer-1 treatment (1 μM) (c). Scale bar, 100 μm; (d) Effects on neuron cell viability following OGD: treatment with the ferroptosis inhibitor Fer-1 and the indicated ATM inhibitors, including AZD1390, significantly increased cell viability; (e, f) AZD1390 reduced the induction of ferroptosis markers, *SLC7A11*(e) and *PTGS2* (f), in the rat brain slice model of ischemic stroke. Transcript levels of *SLC7A11* (e) and *PTGS2* (f) were measured using qPCR from the rat brain slices. *n* = 6 independent biological replicates; (g) Neuronal cell viability in mouse brain slices increased in a dose-dependent manner with AZD1390 treatment. Each gray point in panels d and g represents the neuronal-viability measurement from one individual hemi-coronal brain slice. Twelve slices were analyzed per condition in each independent experiment, and three independent experiments were performed, resulting in 36 slice-level observations per condition. The slices were obtained from 12 animals per experiment and randomly distributed across treatment conditions. Individual slice-to-animal identities were not retained. (d, e, and g) Data represent the median ± IQR. Kruskal-Wallis test with Dunn’s multiple comparison (*p < 0.05; **p < 0.01; ***p < 0.001, ****p < 0.0001). (f) Data represent the mean ± SEM. One-way ANOVA followed by Dunnett’s multiple comparison test. OGD: oxygen glucose deprivation; Fer-1: Ferrostatin-1; ATM: ataxia-telangiectasia mutated; qPCR: quantitative PCR; IQR: interquartile range; SEM: standard error of the mean; ANOVA: analysis of variance.

**Figure 3.**
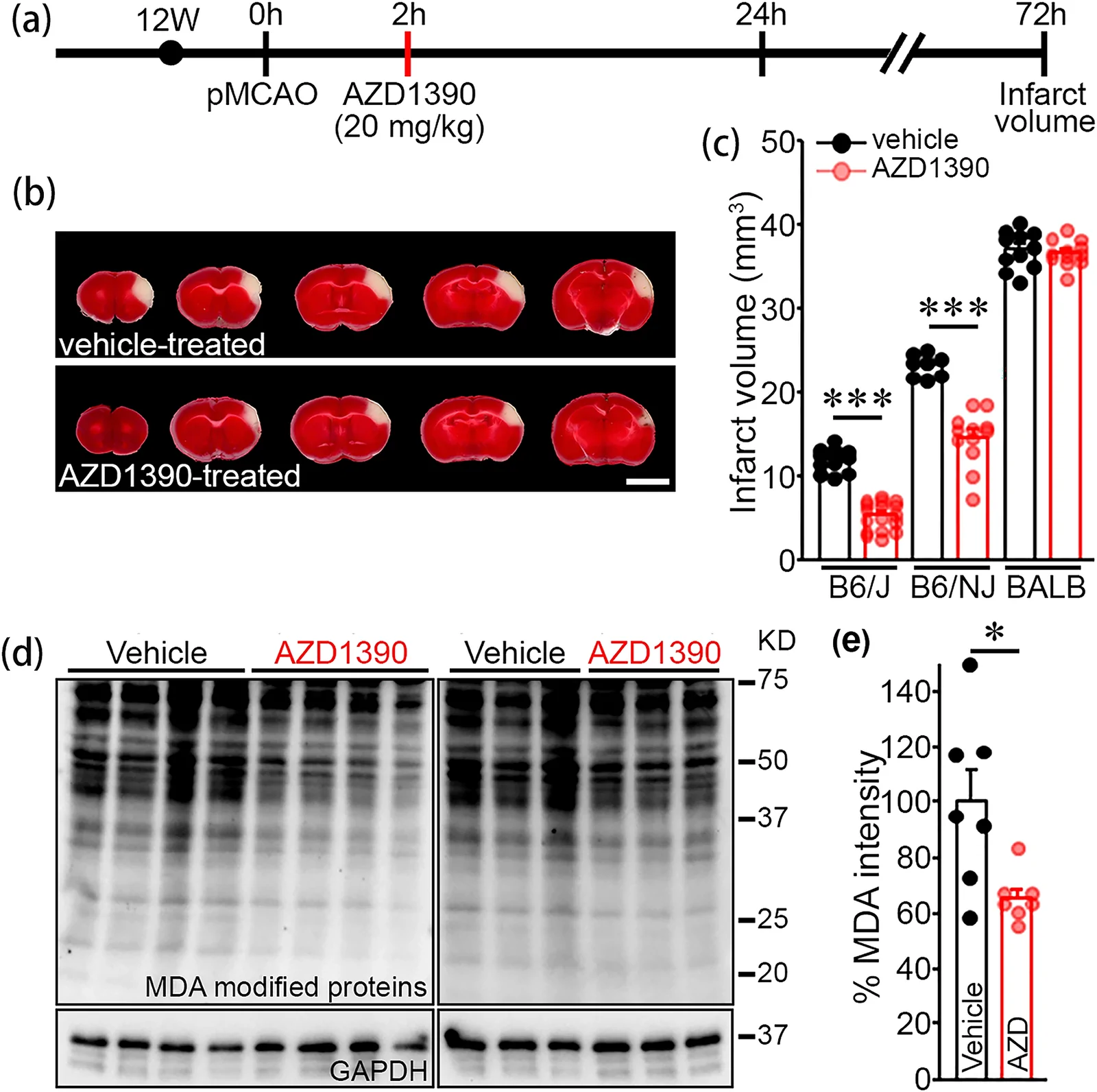
AZD1390 reduces infarct volume after pMCAO in an *in vivo* stroke model. (a) Schematic timeline illustrating the experimental procedure, including ischemic stroke induction through pMCAO and AZD1390 treatment; (b) Serial brain slice sections (1 mm) from the B6/NJ inbred mouse strain. Infarcted tissue appears as white after 2% TTC staining. Scale bar: 5 mm; (c) The graph showing infarct volume 72 hours after pMCAO includes both vehicle-treated control groups (black bar with plots) and AZD1390-treated groups (red bar with plots) for each inbred strain: B6/J, B6/NJ, and BALB. 2-way ANOVA and Šídák’s multiple comparisons (****p < 0.0001); (d) AZD1390 decreased MDA levels in brain tissues from mice subjected to pMCAO followed by vehicle or AZD1390 treatment. The antibody targeting MDA recognized all MDA-modified proteins in brain tissues at different molecular weights. GAPDH was used as a loading control. Each lane represents brain tissue from one individual animal. The vehicle samples are identified as V1-V7, and the AZD1390-treated samples as A1-A7. Samples V1-V4 and A1-A4 were analyzed on one membrane, whereas samples V5-V7 and A5-A7 were analyzed on a second membrane, as indicated by the vertical dividing line. MDA-modified proteins and the corresponding GAPDH loading controls are shown from the same samples. Uncropped images of both membranes, including molecular-weight markers, are provided in Figure S3. Complete sample identities are provided in Table S1; (e) All bands of MDA-modified proteins in each lane were quantified using ImageJ. Quantification was of MDA levels normalized to GAPDH. Data represent the mean ± SEM and statistical significance was determined by two-tailed Student’s *t*-test (*p < 0.05). pMCAO: permanent middle cerebral artery occlusion; TTC: 2,3,5-triphenyltetrazolium chloride; ANOVA: analysis of variance; MDA: malondialdehyde; SEM: standard error of the mean.

**Figure 4.**
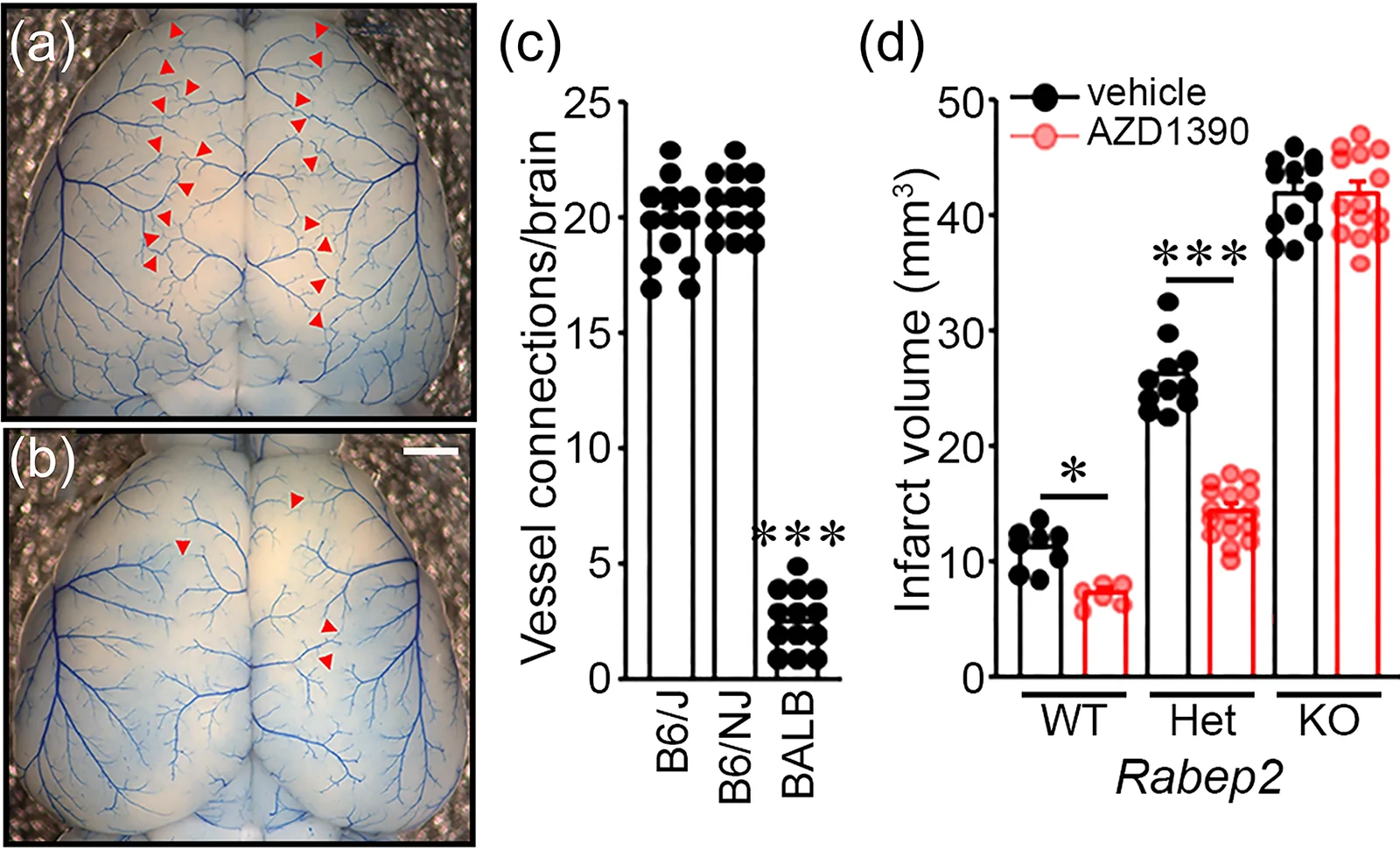
Cerebral collateral vessel density correlates with the therapeutic efficacy of AZD1390 in ischemic stroke. (a, b) Representative images of the brains for inbred mouse strains, B6/NJ (a) and BALB (b). Red arrowheads indicate collateral vessel connections between the ACA and MCA. Scale bar: 1 mm; (c) Average number of collateral vessel connections between the ACA and MCA in the brain. One-way ANOVA followed by Dunnett’s multiple comparison test (***p < 0.001); (d) Infarct volume 72 hours after pMCAO: plot includes both vehicle-treated control groups (black bar with plots) and AZD1390-treated groups (red bar with plots) for *Rabep2* WT, Het, and KO mice. Data represent the mean ± SEM. 2-way ANOVA and Šídák’s multiple comparisons (*p < 0.05, ****p < 0.0001). ACA: anterior cerebral artery; MCA: middle cerebral artery; ANOVA: analysis of variance; pMCAO: permanent middle cerebral artery occlusion; WT: wild-type; Het: heterozygous; KO: knockout; SEM: standard error of the mean.

**Figure 5.**
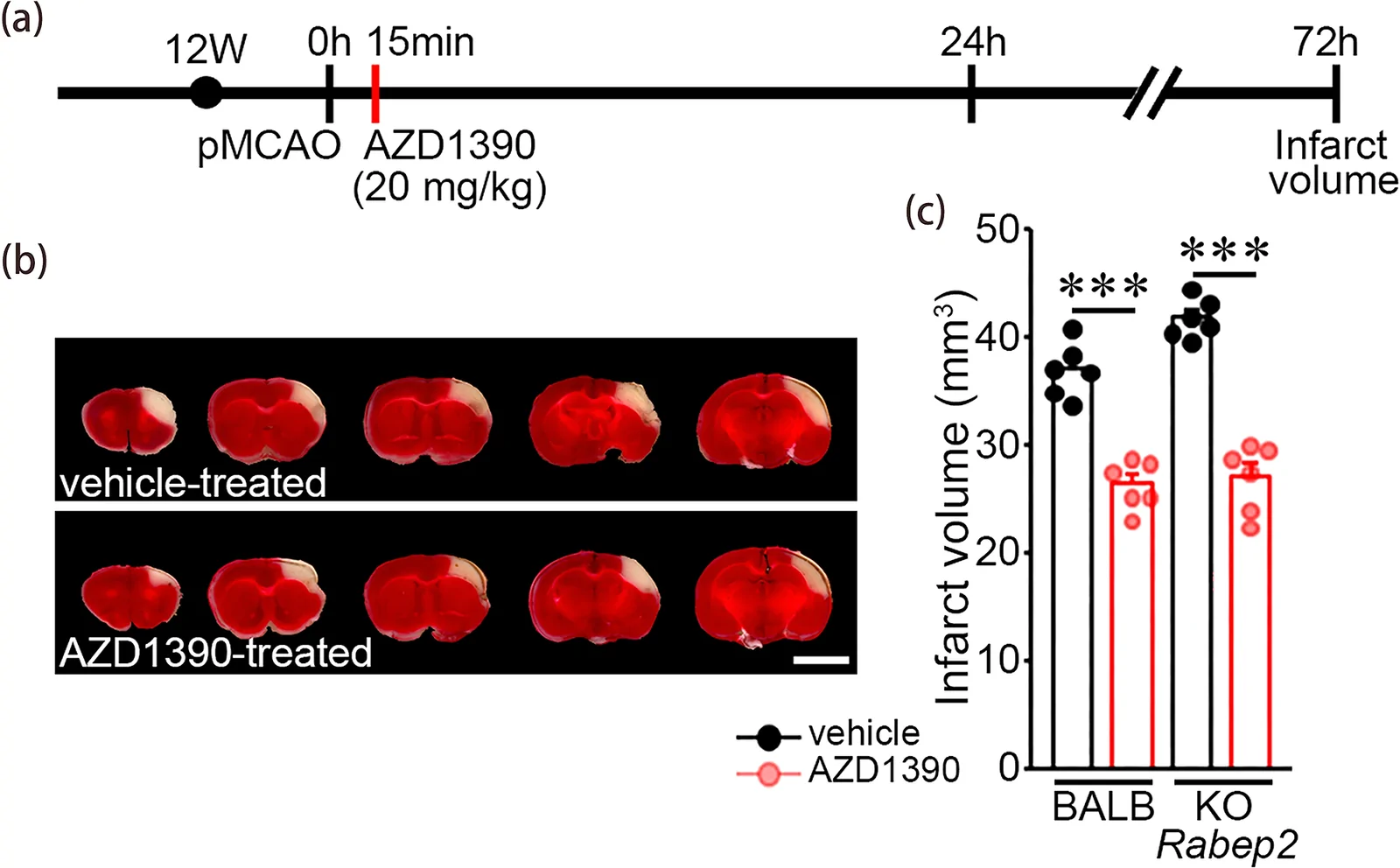
Topical application of AZD1390 rescues stroke-induced brain injury in mice with impaired vascular networks. (a) Schematic of experimental design showing topical application of AZD1390 to the exposed cortex (ischemic territory) 15 minutes after pMCAO, with infarct volume assessed 72 hours post-surgery; (b) Serial brain slice sections (1 mm) from BALB inbred mouse strain. Infarcted tissue appears as white after 2% TTC staining. Scale bar: 5 mm; (c) The graph showing infarct volume 72 hours after pMCAO includes both vehicle-treated control groups (black bar with plots) and AZD1390-treated groups (red bar with plots) for BALB and *Rabep2* KO mice. Data represent the mean ± SEM. 2-way ANOVA and Šídák’s multiple comparisons (*p < 0.05, ****p < 0.0001). pMCAO: permanent middle cerebral artery occlusion; TTC: 2,3,5-triphenyltetrazolium chloride; KO: knockout; SEM: standard error of the mean; ANOVA: analysis of variance.

## Data Availability

The data and materials could be obtained from the corresponding author.
