# Supplementary material for: ATM inhibition attenuates ferroptosis-associated injury and reduces infarct volume in experimental stroke
Source: Ferroptosis Oxid Stress. Author manuscript; Available in PMC 2026 Nov 6. (PMC13730210; doi:10.70401/fos.2026.0046)
Supplement: Supplementary file 1 [file NIHMS2216426-supplement-Supplementary_materials.zip › FOS_202607_Supplementary_material.pdf]

Ferroptosis and Oxidative Stress
https://doi.org/10.70401/fos.2026.0046

Supplementary information

ATM inhibition attenuates ferroptosis-associated injury and reduces infarct volume in experimental stroke

Han Kyu Lee, Chao-Chieh Lin, Denise E. Dunn, Yubin Chen, Ssu-Yu Chen, Douglas A. Marchuk, Scott R. Floyd, Jen-Tsan Chi

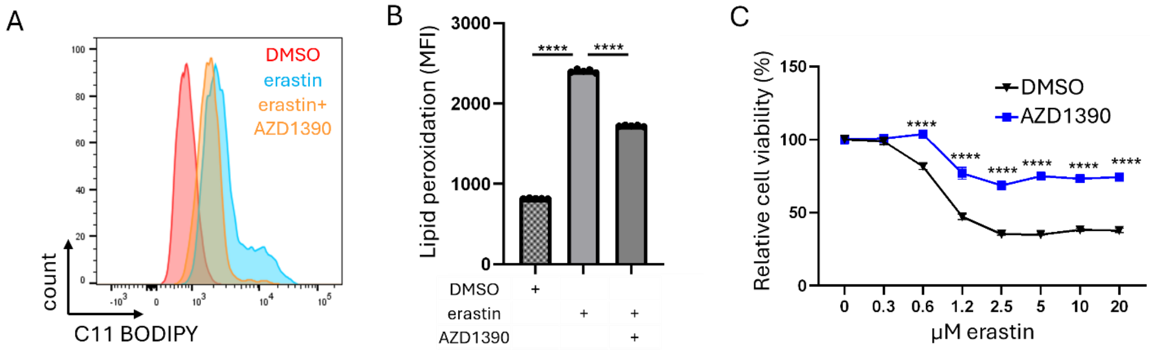

Figure S1. AZD1390 suppresses erastin-induced ferroptosis in HT-22 mouse hippocampal neuronal cells. (A and B) HT-22 cells were treated with erastin (10 mM, 18 h) in the presence or absence of AZD1390 (10 mM). Lipid peroxidation was assessed by C11-BODIPY staining (A) and quantified as mean fluorescence intensity in the oxidized C11-BODIPY-positive cells (B). Data are presented as mean ± SEM from n = 5 independent biological replicates. Statistical significance was determined by one-way ANOVA followed by Dunnett’s multiple-comparisons test (**** $p < 0.0001$). (C) HT-22 cells were treated with increasing concentrations of erastin (24 h) in the presence or absence of AZD1390 (10 mM), and cell viability was measured using the CellTiter-Glo assay. Data are presented as mean ± SEM. Statistical significance was determined by two-way ANOVA followed by Dunnett's multiple-comparisons test (**** $p < 0.0001$).

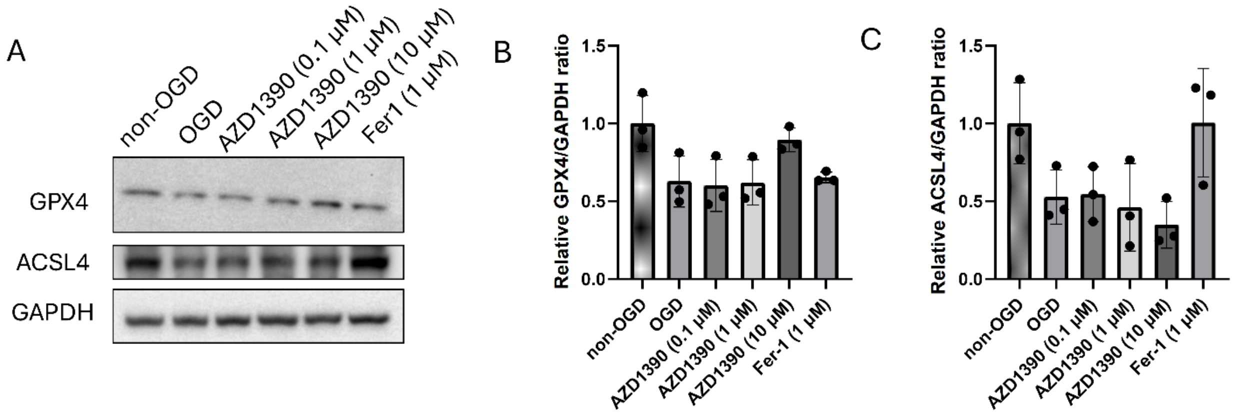

Figure S2. AZD1390 does not alter the expression of ferroptosis-associated proteins in the ex vivo cortical brain slice ischemia model. Rat cortical brain slices were subjected to oxygen-glucose deprivation (OGD) and treated with the indicated concentrations of AZD1390 (0.1, 1, or 10 μM) or ferrostatin-1 (Fer-1, 1 μM). (A) Representative immunoblots of GPX4, ACSL4, and GAPDH. (B and C) Densiometric quantification of GPX4 (B) and ACSL4 (C) using ImageJ. Protein levels were normalized to GAPDH and expressed relative to the non-OGD control. Data are presented as mean ± SEM from n = 3 independent biological replicates. Statistical significance was assessed using the Kruskal-Wallis test; no statistically significant differences were detected among the groups.

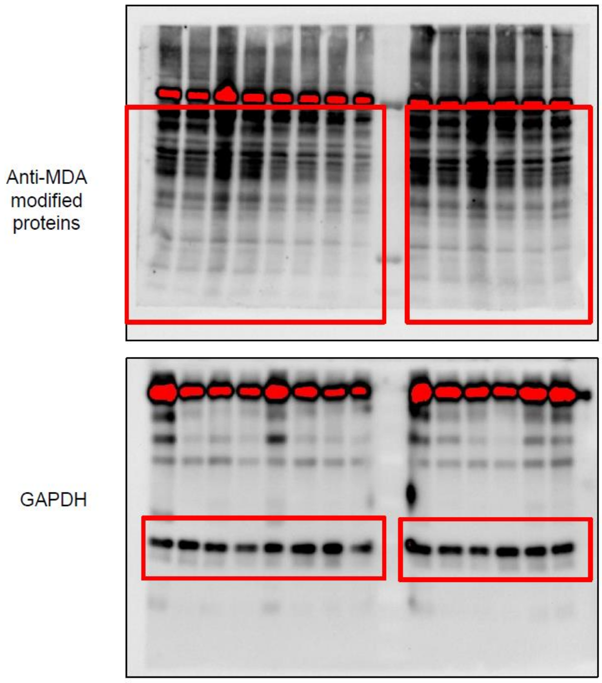

Figure S3. Full unedited blot/gel for Figure 3D.
